# Supplementary material for: Cortical ignition dynamics is tightly linked to the core organisation of the human connectome
Source: PLoS Comput Biol. 2020 Jul 31;16(7):e1007686. doi: 10.1371/journal.pcbi.1007686 (PMC7423150; doi:10.1371/journal.pcbi.1007686)
Supplement: S1 File — Supplemental Figures A-F and Supplemental Tables A-B. (PDF) [file pcbi.1007686.s001.pdf]

Supporting information 1 for: Cortical ignition dynamics is tightly linked to the core organization of the human connectome

Samy Castro[1,2,*], Wael El-Deredy[3], Demian Battaglia[4,#] and Patricio Orio[1,#]

[1] *Centro Interdisciplinario de Neurociencias de Valparaíso, Universidad de Valparaíso, Valparaíso, Chile*

[2] *Programa de Doctorado en Ciencias, mención Neurociencia, Universidad de Valparaíso, Valparaíso, Chile.*

[3] *Centro de Investigación y Desarrollo en Ingeniería en Salud, Universidad de Valparaíso, Valparaíso, Chile*

[4] *Aix-Marseille Université, Institut de Neurosciences des Systèmes, INSERM UMR 1106, Marseille, France*

[*] First authorship; [#] Shared last authorship

E-mail: patricio.orio@uv.cl (PO); demian.battaglia@univ-amu.fr (DB)

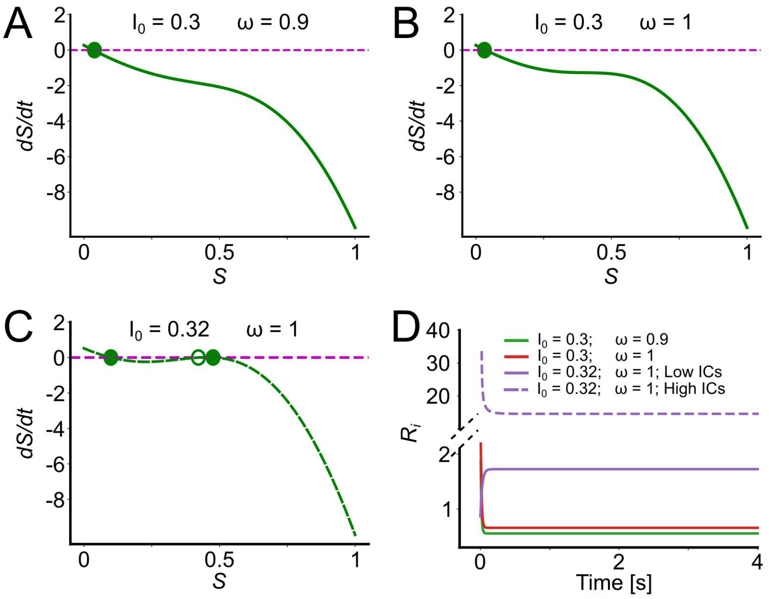

Fig A. Phase portrait and time-series of an isolated cortical area using the mean-field model.

(A-C) Three solutions as a function of I_0 and ω for an isolated node (**A** (I_0=0.3; ω=0.9), **B** (I_0=0.3; ω=1), and C (I_0=0.32; ω=1)). The dynamics of the isolated node gets two attractors in **C**. **(D)** Mean firing rate (*y-axis*) in time (*x-axis*) for the three sets of parameters. In *purple* (the isolated region with two attractors), segmented and solid lines are simulations started from High ICs (0.9≤Si≤1) or Low ICs (0≤Si≤0.01), respectively. The mean-field model (MFM) with a single attractor (*green* and *red*) only displays a low mean firing rate.

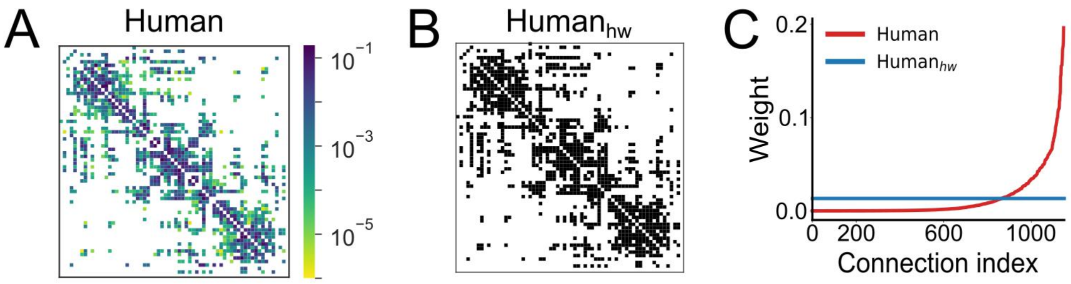

Fig B. The *$Human_{hw}$* connectome.

(A-B) The structural connectivity matrix of **(A)** *Human* and **(B)** *$Human_{hw}$* (with homogeneous weights in its connections). **(C)** Sorted connection weights of *Human* (*red*) and *$Human_{hw}$* (*blue*) connectomes. Note the structural heterogeneity in the *Human*. Both connectomes have 1148 connections, and 15.3 of overall strength.

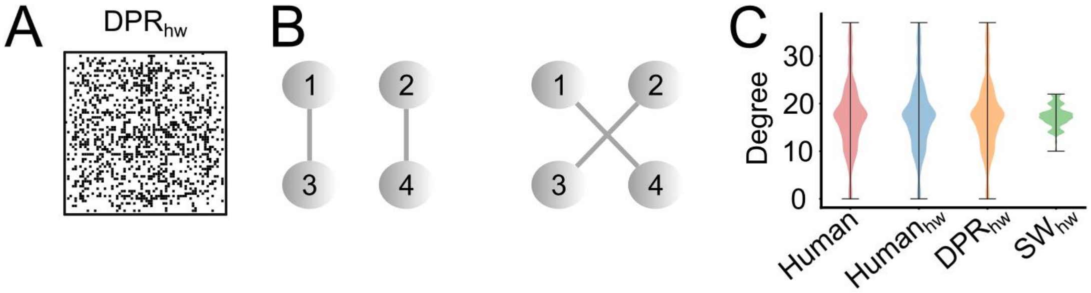

Fig C. The DPR_{hw} connectome.

(A) One representative example of the Degree-Preserving Random (DPR_{hw}) structural connectivity matrix with homogeneous weights. **(B)** An illustrative example of the Maslov & Sneppen algorithm, adapted from Fornito et al., 2016. The algorithm was applied one hundred times to the *Human* to build the DPR_{hw} connectomes. **(C)** Degree distribution of *Human*, $Human_{hw}$, DPR_{hw}, and SW_{hw}. DPR_{hw} connectomes have the same degree distribution, the number of connections (1148), and overall strength (15.3) of the *Human*.

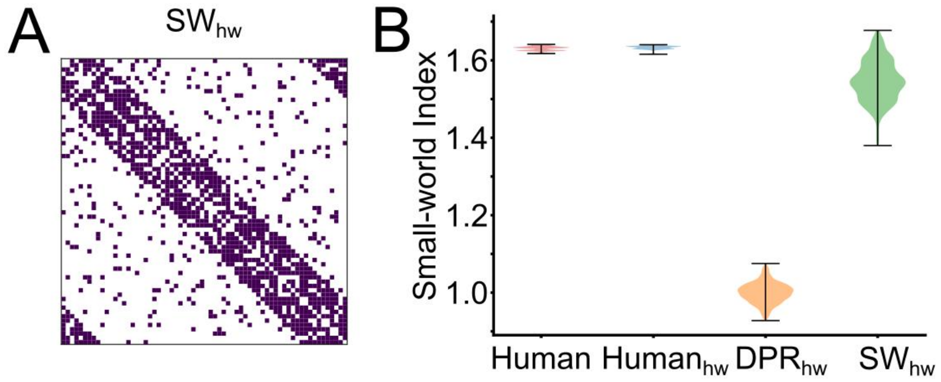

Fig D. The SW_{hw} connectome.

(A) One representative example of the Small-World (SW_{hw}) structural connectivity matrix with homogeneous weights. **(B)** Small-world index of *Human*, $Human_{hw}$, DPR_{hw}, and SW_{hw}. Note that the precise value of the small-world index depends on the random network used to normalize. Thus, its value is not deterministic, as shown by the error bars of the *Human*. The SW_{hw} connectomes conserve the number of connections (1148), and overall strength (15.3) of the *Human*.

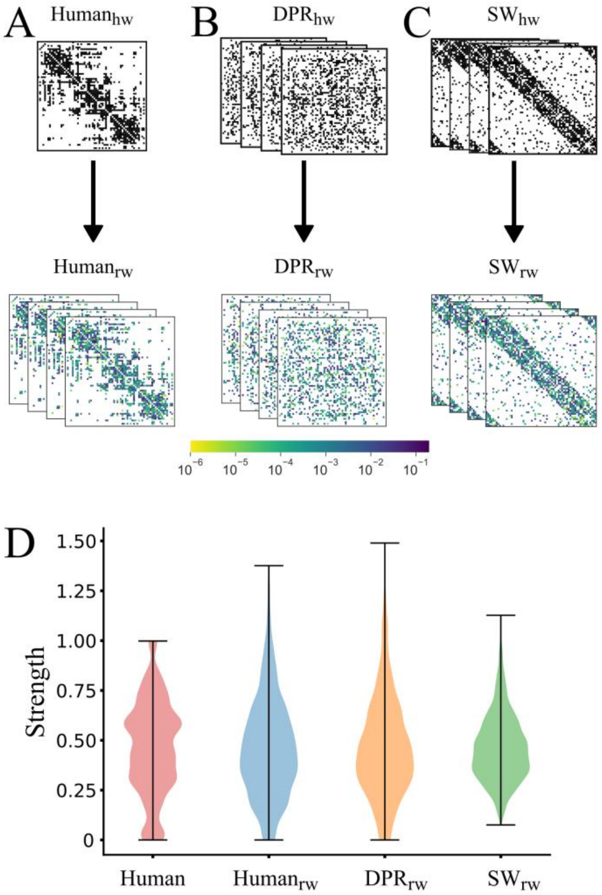

Fig E. The weighted surrogate models conserve the weight distribution of the *Human*.

The values were assigned by random permutation of *Human* connections to create: **(A)** 60 $Human_{rw}$ from $Human_{hw}$; **(B)** 60 DPR_{rw}, from 60 DPR_{hw}; and **(C)** 60 SW_{rw}, from 60 SW_{hw}. **(D)** Strength distribution of *Human*, $Human_{rw}$, DPR_{rw}, and SW_{rw}.

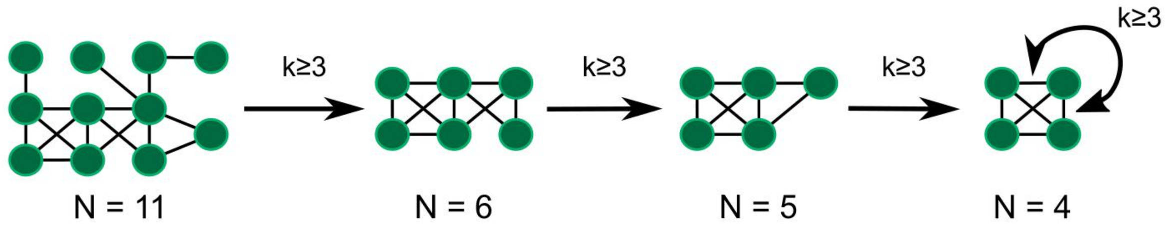

Fig F. Diagram of the k-core *decomposition* algorithm.

The *k*-core decomposition is used to extract the unweighted core nodes in the networks. In each step, the nodes (N) that have a degree< k_i are removed in successive steps until the sub-set remains constant. The four core nodes are interconnected with ki ≥3 and thus this constitutes the 3-core. In the case of weighted networks, the s-core decomposition extracts the core nodes based on their strength (≤ si).

Table A. Abbreviations and names of cortical areas from Desikan-Killiany atlas. Adapted from Hansen et al., 2015.

Abbreviation	Cortical Area
**ENT**	Enthorinal cortex
**PARH**	Parahippocampal cortex
**TP**	Temporal pole
**FP**	Frontal pole
**FUS**	Fusiform gyrus
**TT**	Transverse temporal cortex
**LOCC**	Lateral occipital cortex
**SP**	Superior parietal cortex
**IT**	Inferior temporal cortex
**IP**	Inferior temporal cortex
**SMAR**	Supramarginal gyrus
**BSTS**	Bank of the superior temporal sulcus
**MT**	Middle temporal cortex
**ST**	Superior temporal cortex
**PSTC**	Postcentral gyrus
**PREC**	Precentral gyrus
**CMF**	Caudal middle frontal cortex
**POPE**	Pars opercularis
**PTRI**	Pars triangularis
**RMF**	Rostral middle frontal cortex
**PORB**	Pars orbitalis
**LOF**	Lateral orbitofrontal cortex
**CAC**	Caudal anterior cingulate cortex
**RAC**	Rostral anterior cingulate cortex
**SF**	Superior frontal cortex
**MOF**	Medial orbitofrontal cortex
**LING**	Lingual gyrus
**PCAL**	Pericalcarine cortex
**CUN**	Cuneus
**PARC**	Paracentral lobule
**ISTC**	Isthmus of the cingulate cortex
**PCUN**	Precuneus
**PC**	Posterior cingulate cortex

Table B. The identity of the ignited cortical areas at the bifurcation point G_-.

Hagmann dataset, based on DKA parcellation has 11 cortical areas ignited (Hagmann et al., 2008). Schriner dataset, based on DKA parcellation has 12 cortical areas ignited (Schriner et al., 2015). Deco dataset (Deco et al., 2018), based on AAL parcellation (Rolls et al., 2015) has 9 cortical areas ignited. Left **(L)** and Right (**R**) hemisphere.

Hagmann dataset	Schriner dataset	Deco dataset
**Pericalcarine Cortex<br>(PCAL)<br>L / R**	**Pericalcarine Cortex<br>(PCAL)<br>R**	**-**
**Cuneus<br>(CUN)<br>L / R**	**-**	**-**
**Paracentral Lobule<br>(PARC)<br>L**	**Paracentral Lobule<br>(PARC)<br>R**	**-**
**Isthmus of the Cingulate Cortex<br>(ISTC)<br>L / R**	**Isthmus of the Cingulate Cortex<br>(ISTC)<br>L**	**-**
**Precuneus<br>(PCUN)<br>L / R**	**-**	**Precuneus<br>(PCUN)<br>R**
**Posterior Cingulate Cortex<br>(PC)<br>L / R**	**-**	**-**
**-**	**Temporal pole<br>(TP)<br>R**	**-**
**-**	**Lateral Occipital Cortex<br>(LOCC)<br>R**	**-**
**-**	**Pars Orbitalis<br>(PORB)<br>R**	**-**
**-**	**Caudal Anterior Cingulate Cortex<br>(CAC)<br>R**	**-**
**-**	**Lingual Gyrus<br>(LING)**	**-**

	**L**	
**-**	**Supramarginal Gyrus (SMAR) L**	**Supramarginal Gyrus (SMG) R**
**-**	**Middle Temporal Cortex (MT) L**	**-**
**-**	**Transverse Temporal Cortex (TT) L**	**-**
**-**	**Frontal Pole (FP) L**	**-**
**-**	**-**	**Posterior Cingulate Gyrus (PCC) L**
**-**	**-**	**Parahippocampal Gyrus (PHG) L**
**-**	**-**	**Hippocampus (HIP) L**
**-**	**-**	**Calcarine Fissure (CAL) L**
**-**	**-**	**Inferior Parietal Gyrus (IPG) L**
**-**	**-**	**Inferior Temporal Gyrus (ITG) R**
**-**	**-**	**Angular Gyrus (ANG) R**
