# Supplementary material for: Cortical ignition dynamics is tightly linked to the core organisation of the human connectome
Source: PLoS Comput Biol. 2020 Jul 31;16(7):e1007686. doi: 10.1371/journal.pcbi.1007686 (PMC7423150; doi:10.1371/journal.pcbi.1007686)
Supplement: S2 File — Supplemental Figures G-N. (PDF) [file pcbi.1007686.s002.pdf]

Supporting information 2 for: Cortical ignition dynamics is tightly linked to the core organization of the human connectome

Samy Castro[1,2,*], Wael El-Deredy[3], Demian Battaglia[4,#] and Patricio Orio[1,#]

[1] Centro Interdisciplinario de Neurociencias de Valparaíso, Universidad de Valparaíso, Valparaíso, Chile

[2] Programa de Doctorado en Ciencias, mención Neurociencia, Universidad de Valparaíso, Valparaíso, Chile.

[3] Centro de Investigación y Desarrollo en Ingeniería en Salud, Universidad de Valparaíso, Valparaíso, Chile

[4] Aix-Marseille Université, Institut de Neurosciences des Systèmes, INSERM UMR 1106, Marseille, France

[*] First authorship; [#] Shared last authorship

E-mail: patricio.orio@uv.cl (PO); demian.battaglia@univ-amu.fr (DB)

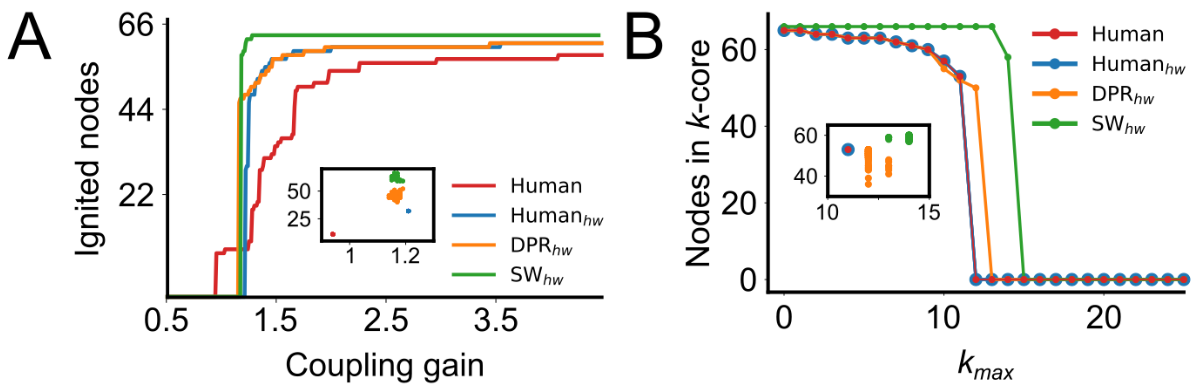

Fig G. The ignited nodes and the *k*-core decomposition of the `Human`.

(A) Number of ignited nodes (*y-axis*) (threshold $R_i > 5$), as a function of coupling gain G (*x-axis*) in $\mathtt{Human}$ (*red*), $\mathtt{Human}_{hw}$ (*blue*), one example of $\mathtt{DPR}_{hw}$ (*orange*), and one example of $\mathtt{SW}_{hw}$ (*green*). The inset shows the number of ignited at the G_- bifurcation, for $\mathtt{Human}$ and all the individual uSCs. **(B)** *k*-core decomposition of $\mathtt{Human}$, $\mathtt{Human}_{hw}$, $\mathtt{DPR}_{hw}$, and $\mathtt{SW}_{hw}$. The *y-axis* shows the number of nodes in the shell, whereas the *x-axis* shows the minimum k_{max} inside that shell. The inset shows the largest k_{max}, k_{max}-core, for $\mathtt{Human}$ and all the uSCs. The $\mathtt{Human}$ has the lowest degree (k_{max} =11) and largest k_{max}-core (53 regions, ~80.3% of network nodes).

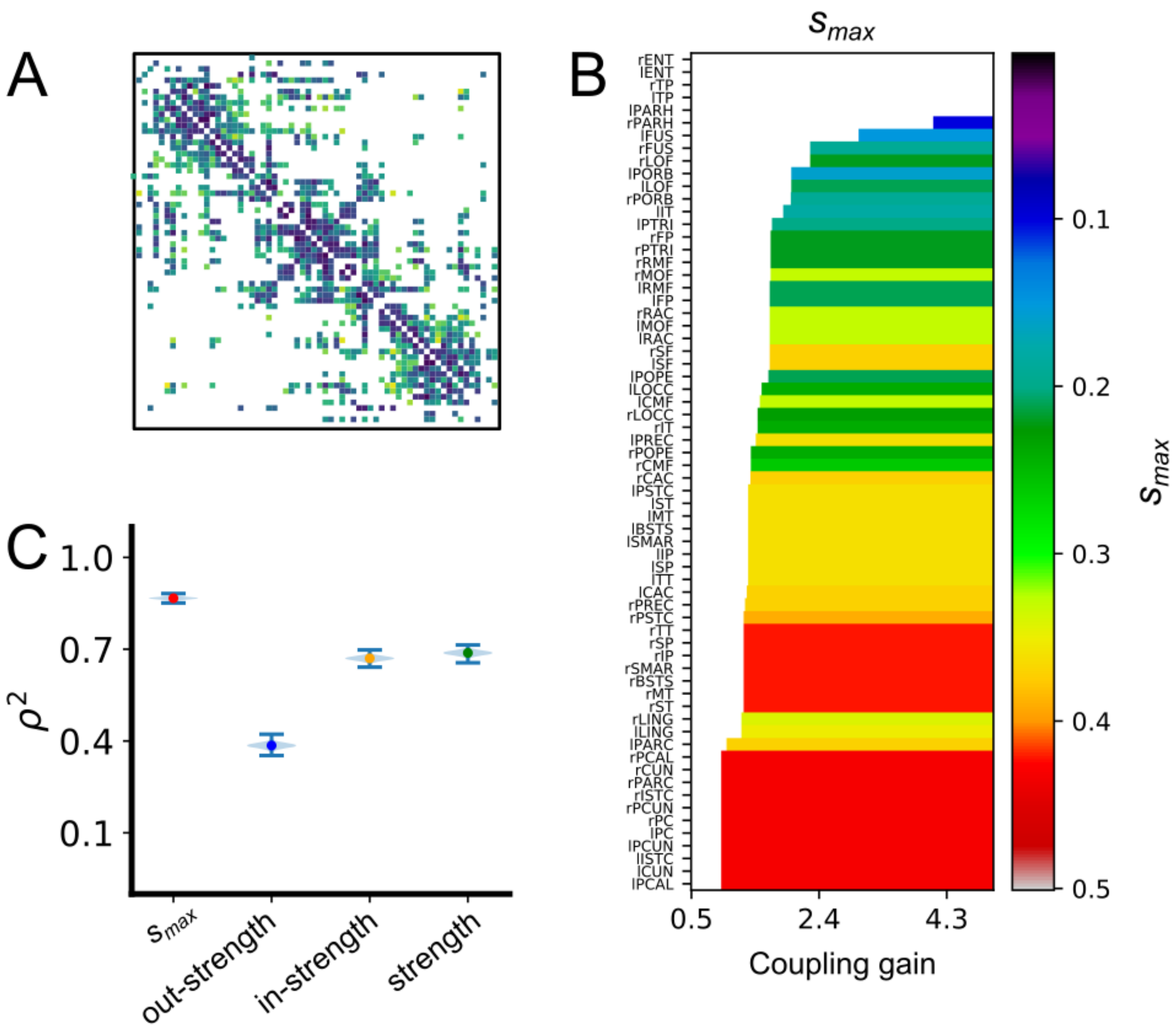

Fig H. The ignition, s_{max} and strength levels for each node in the *Human* connectome.

(A) The *Human* connectivity matrix. **(B)** The cortical areas (*y-axis*) were sorted by the coupling gain (*x-axis*) in which their first ignite. The colorbar shows the s_{max} value of each cortical area. **(C)** Spearman rank correlation squared (ρ^2, shared variance) between the *G* value at ignition of each node and its s_{max} (**0.867**); out-strength (**0.386**); in-strength (**0.671**), and strength (**0.687**). The significance of ρ^2 was evaluated using 10.000 replicas from bootstrap resampling (the blue violin plots).

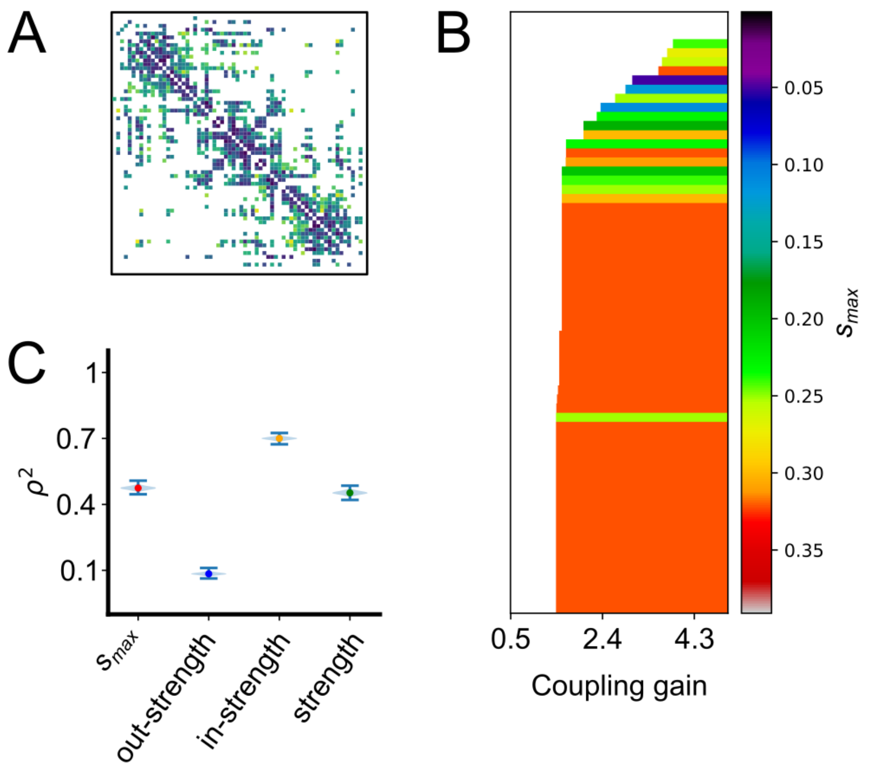

Fig J. The ignition, s_{max} and strength levels for each node in the $Human_{rw}$ connectome.

(A) The $Human_{rw}$ connectivity matrix. **(B)** The cortical areas (*y-axis*) were sorted by the coupling gain (*x-axis*) in which their first ignite. The colobar shows the s_{max} value of each cortial area when were ignited. **(C)** Spearman rank correlation squared (ρ^2, shared variance) between the *G* value at ignition of each node and its s_{max} (**0.474**); out-strength (**0.084**); in-strength (**0.700**), and strength (**0.453**). The significance of ρ^2 was evaluated using 10.000 replicas from bootstrap resampling (the blue violin plots).

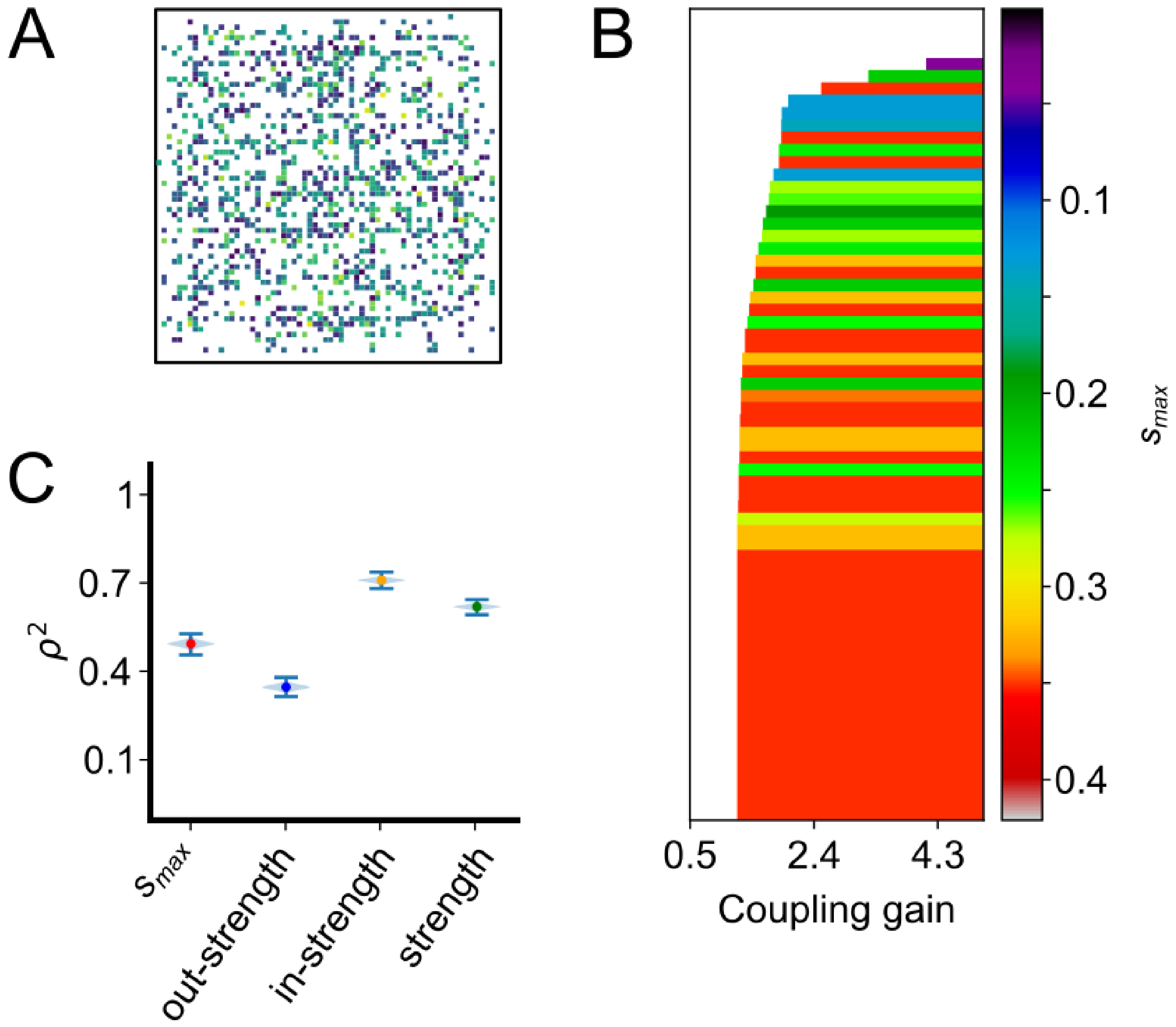

Fig K. The ignition, s_{max} and strength levels for each node in the DPR_{rw} connectome.

(A) The DPR_{rw} connectivity matrix. **(B)** The cortical areas (*y-axis*) were sorted by the coupling gain (*x-axis*) in which their first ignite. The colorbar shows the s_{max} value of each cortical area when were ignited. **(C)** Spearman rank correlation squared (ρ^2, shared variance) between the G value at ignition of each node and its s_{max} (**0.495,** *red*); out-strength (**0.348,** *blue*); in-strength (**0.709,** *yellow*), and strength (**0.620**, *green*). The significance of ρ^2 was evaluated using 10.000 replicas from bootstrap resampling (the blue violin plots).

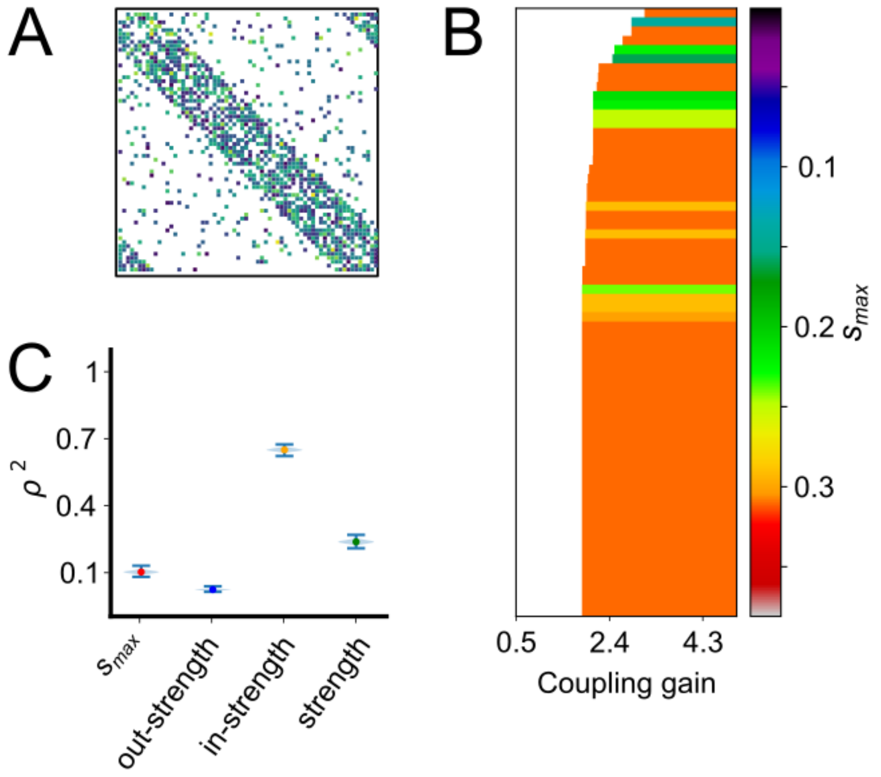

Fig L. The ignition, s_{max} and strength levels for each node in the SW_{rw} connectome.

(A) The SW_{rw} connectivity matrix. **(B)** The cortical areas (*y-axis*) were sorted by the coupling gain (*x-axis*) in which their first ignite. The colobar shows the s_{max} value of each cortial area when were ignited. **(C)** Spearman rank correlation squared (ρ^2, shared variance) between the *G* value at ignition of each node and its s_{max} (**0.100**); out-strength (**0.021**); in-strength (**0.650**), and strength (**0.235**). The significance of ρ^2 was evaluated using 10.000 replicas from bootstrap resampling (the blue violin plots).

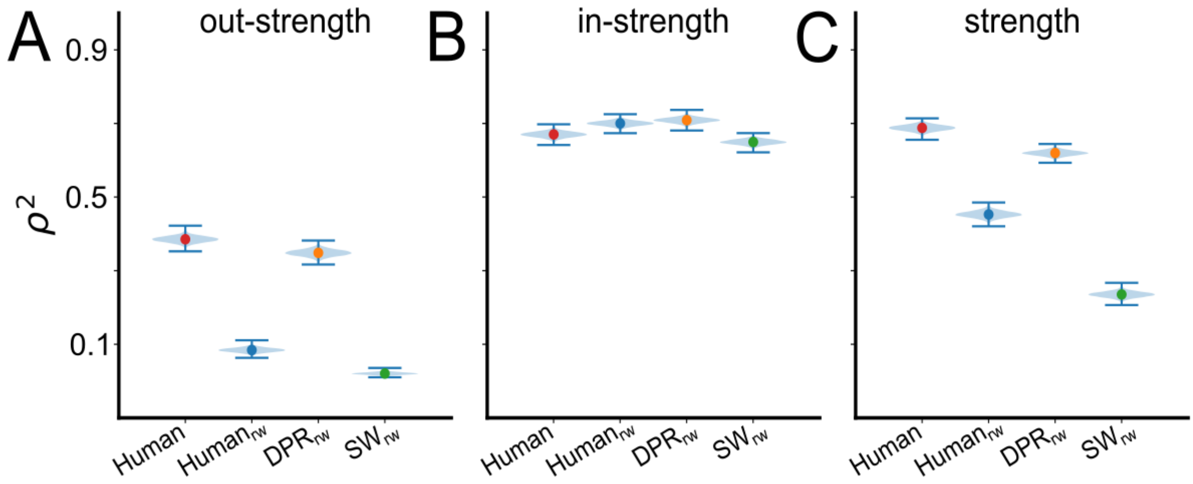

Fig M. Correlation between ignition order and strength for *Human* and wSCs.

(A) Spearman rank correlation squared (ρ^2, shared variance) between the G value at the ignition of each node and its out-strength in *Human* (**0.386**), $Human_{rw}$ (**0.084**), DPR_{rw} (**0.348**), and SW_{rw} (**0.021**). **(B)** Spearman rank correlation squared (ρ^2, shared variance) between the G value at the ignition of each node and its in-strength in *Human* (**0.671**), $Human_{rw}$ (**0.700**), DPR_{rw} (**0.709**), and SW_{rw} (**0.650**). **(C)** Spearman rank correlation squared (ρ^2, shared variance) between the G value at ignition of each node and its strength in *Human* (**0.687**), $Human_{rw}$ (**0.453**), DPR_{rw} (**0.620**), and SW_{rw} (**0.235**). The significance of ρ^2 was evaluated using 10.000 replicas from bootstrap resampling (the blue violin plots).

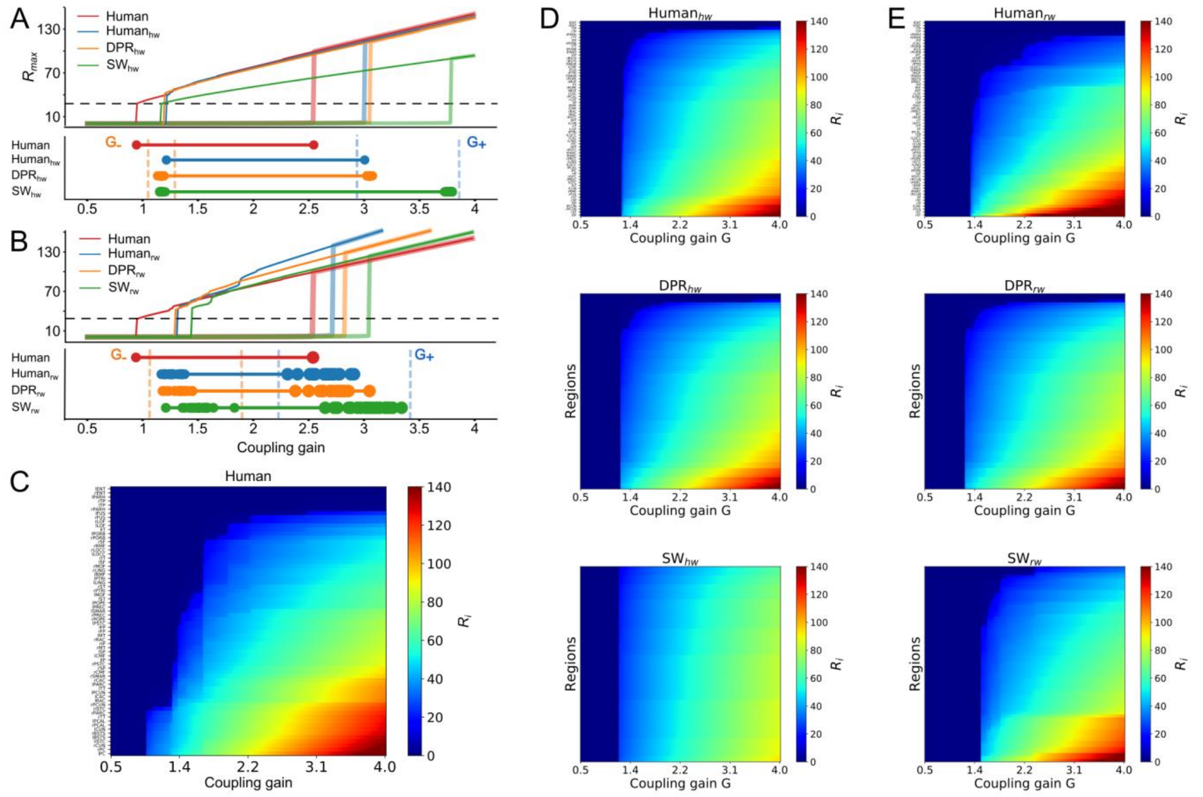

Fig N. Overall Rmax and Ri per region of the Human, uSCs, and wSCs ensembles.

(A-B) Top, R_{max} versus coupling gain G in simulation starting either from High ICs (0.3≤Si≤1) or Low ICs (0≤Si≤0.1) for **(A)** Human and uSCs, and **(B)** Human and wSCs. At the bottom, the bifurcation points G_- and G_+ for the corresponding ensembles. **(C-E)** Mean firing rate activity, R_i (colour bar) of each region *i* (*y-axis*) versus coupling gain G (*x-axis*) for the **(C)** Human, **(D)** uSCs ensembles, and **(E)** wSCs ensembles. The simulations started from High ICs (0.3≤Si≤1).
