# Supplementary material for: Cortical ignition dynamics is tightly linked to the core organisation of the human connectome
Source: PLoS Comput Biol. 2020 Jul 31;16(7):e1007686. doi: 10.1371/journal.pcbi.1007686 (PMC7423150; doi:10.1371/journal.pcbi.1007686)
Supplement: S3 File — (PDF) [file pcbi.1007686.s003.pdf]

Supporting information 3 for: Cortical ignition dynamics is tightly linked to the core organization of the human connectome

Samy Castro[1,2,*], Wael El-Deredy[3], Demian Battaglia[4,#] and Patricio Orio[1,#]

[1] *Centro Interdisciplinario de Neurociencias de Valparaíso, Universidad de Valparaíso, Valparaíso, Chile*

[2] *Programa de Doctorado en Ciencias, mención Neurociencia, Universidad de Valparaíso, Valparaíso, Chile.*

[3] *Centro de Investigación y Desarrollo en Ingeniería en Salud, Universidad de Valparaíso, Valparaíso, Chile*

[4] *Aix-Marseille Université, Institut de Neurosciences des Systèmes, INSERM UMR 1106, Marseille, France*

[*] First authorship; [#] Shared last authorship

E-mail: patricio.orio@uv.cl (PO); demian.battaglia@univ-amu.fr (DB)

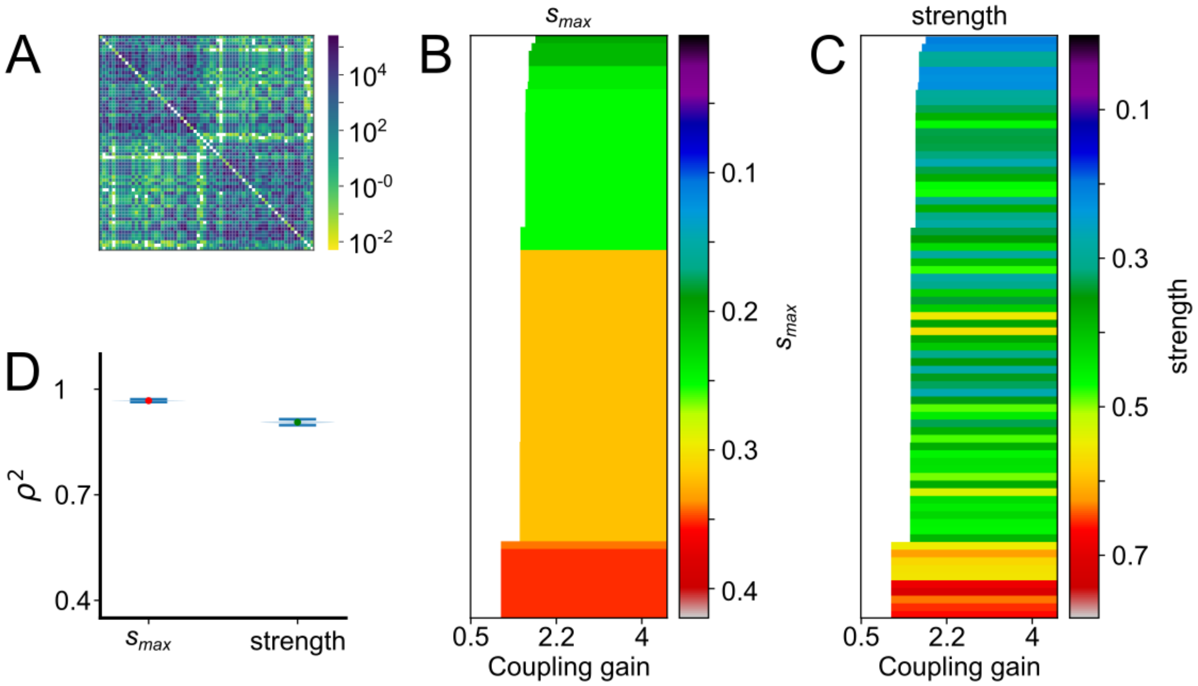

Fig A. The ignition, s_{max} and strength levels for each node in the *Schriner* dataset.

(A) The *Schriner* connectivity matrix was parcellated using the Desikan-Killiany atlas (66 regions and 4290 connections) (Schriner et al., 2015). (B) Relationship between ignition and s_{max} of each cortical region of the Schriner connectome. Cortical regions in the y-axis are sorted according to the coupling gain G (x-axis) value at which they first ignite. Colour code shows the smax for each of the ignited cortical regions. (C) Similarly, relationship between ignition recruitment and the strength (color bar) of each cortical region. (D) Explained variance of Spearman rank correlation (ρ2) between ignition recruitment and the smax (0.**967**, red dot), and strength (0.906, green dot). The Schriner connectome shows that s_{max} has a significantly higher explained variance of ignition recruitment than the strength. However, both rank values are very high. The significance of ρ^2 was evaluated using 10.000 replicas from bootstrap resampling (violin plots).

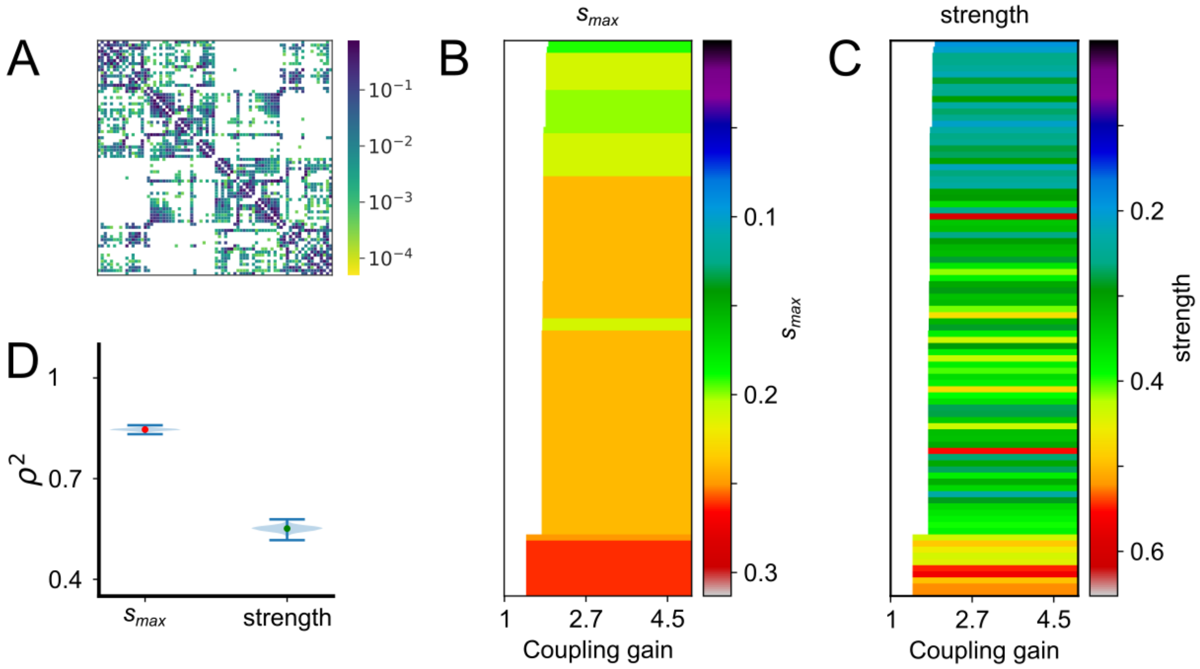

Fig B. The ignition, s_{max} and strength levels for each node in the *Deco* dataset.

(A) The `Deco` connectivity matrix was parcellated using the Automated Anatomical Labelling atlas (76 regions and 2076 connections) (Deco et al., 2018). **(B)** Cortical areas (*y-axis*) were sorted by the coupling gain (*x-axis*) at which their first ignite. The colour bar shows the s_{max} value of each cortical area when were ignited. (C) Relationship between ignition recruitment and the strength (color bar) of each cortical region. (D) Explained variance of Spearman rank correlation (ρ2) between ignition recruitment and the s_{max} (0.846), and strength (0.551). The significance of ρ^2 was evaluated using 10.000 replicas from bootstrap resampling (the blue violin plots).

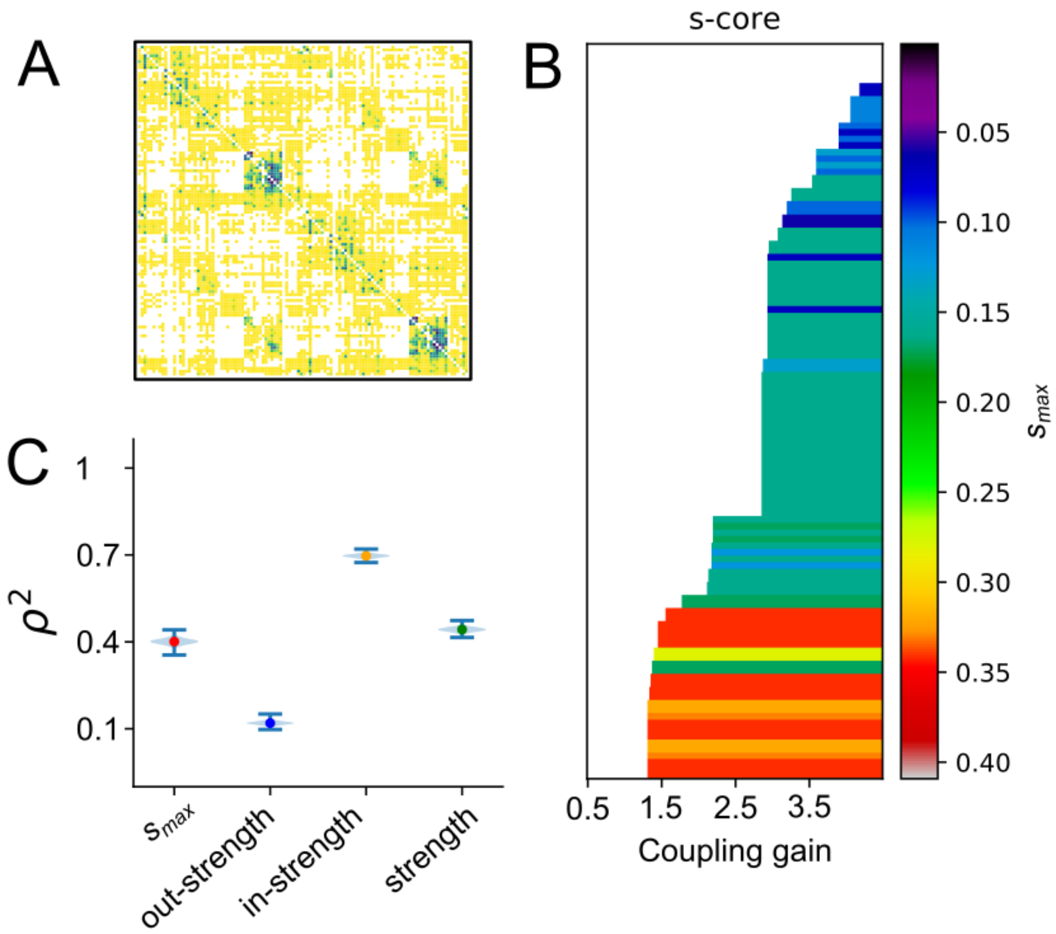

Fig C. The ignition, s_{max} and strength levels for each node in the `Mouse` (*Mus musculus*) dataset.

(A) The `Mouse` connectivity matrix contains 112 regions (56 per hemisphere) and 6542 connections. **(B)** The cortical areas (*y-axis*) were sorted by the coupling gain (*x-axis*) in which their first ignite. The colorbar shows the s_{max} value of each cortical area when were ignited. **(C)** Spearman rank correlation squared (ρ^2, shared variance) between the G value at ignition of each region and its s_{max} (**0.761**) and out-strength (**0.246**), in-strength (**0.913**), and strength (**0.673**). The significance of ρ^2 was evaluated using 10,000 replicas from bootstrap resampling (the blue violin plots).
