# Supplementary material for: Cortical ignition dynamics is tightly linked to the core organisation of the human connectome
Source: PLoS Comput Biol. 2020 Jul 31;16(7):e1007686. doi: 10.1371/journal.pcbi.1007686 (PMC7423150; doi:10.1371/journal.pcbi.1007686)
Supplement: Attachment — Submitted filename: Reply to reviewers.pdf [file pcbi.1007686.s004.pdf]

We are grateful for the constructive comments and concerns expressed by the reviewers, they helped to make our manuscript better and to identify very interesting issues. A detailed response to the issues risen by reviewers follows.
Before, we have to point out a mistake we detected in one of our analyses. In Figure 5C, where we dissect the contribution of in-strengths versus out-strengths to the ignition core, we had inadvertently swapped the in and out strengths. In the first version of the manuscript we found a better correlation between out-strengths and ignition order, but it was actually in-strength as it is, incidentally, easier to understand. It is important to stress that this confusion does not affect any of our main results, since they do not depend on the directedness of the graph or are invariant with respect to interchanging in- and out-strengths. We have now accordingly corrected the figure labels and captions and the few sentences in the Results and Discussion sections referring to it.
Additionally, we fixed several minor errors that we detected and are properly highlighted in the version with changes tracked.

Reviewer #1: In this work, Castro and colleagues explore the factors favouring intrinsic ignition in brain activity using a mean-field whole-brain modelling approach by varying the strength of effective inter-regional coupling gain. […]

The paper is very well written, with the introduction covering the most relevant literature and the discussion covering the most important points and scenarios. The choice of the model is adequately justified to test the hypothesis in question and the authors demonstrate effort in making clear and illustrative Figures of the methods and results.

We wish to thank the reviewer for her/his appreciation of our study and presentation.

Although the paper is publishable in its current form, I have a few suggestions to improve the quality of the manuscript.

<u>Comments/Corrections/Suggestions:</u>

Abstract:
✓weighed -> weighted

Response: Fixed

✓Last word of the abstract: dynamic -> dynamics (dynamic is an adjective, dynamics is the noun)

Response: Fixed

Summary:
✓Corresponds with -> corresponds to

Response: The whole sentence has been re-written

Introduction:
✓Growing […] evidence stress -> stresses

Response: Fixed

The authors describe the results of the work directly in the introduction (page 5 and 6), which is not common practice. I suggest moving this to the discussion section and keeping the introduction to introduce the hypothesis that will be verified by the model. On the other hand, in the results section, the authors comment on the facts justifying the hypothesis, which could instead be moved to the introduction, i.e.:
in page 8: *'When moving from regional to whole-brain network dynamics, we can expect, in agreement with several authors [6,13,18,26,27] that the spontaneous ignition dynamics and state shifting in different regions will be shaped by the underlying structural connectivity (SC) included in the model.'*
In page 10: *'The human connectome is associated with specific distributions of the local organisation, such as node degrees (i.e. the number of neighbouring regions) or node in- or out-strengths (i.e. the sum of the weights of incoming or outgoing connections), as well as of global organisation such as small-worldness [29,30]. It is not clear a priori how these different specific levels of organisation of the connectome influence the ignition behaviour of mean field models built on them. Therefore, to test the relevance of the Human connectome (Human) organisation in determining the ignition behaviour, we compared the simulated dynamics of a mean-field model based on the Human, with alternative surrogate connectomes. The surrogate connectomes conserve key features of Human organisation while selectively randomising others.'*

Response: We appreciate the suggestion. We have reduced the outline of results (almost to one half) but nevertheless we are keeping some part of it, as we feel that it is good for the reader to have an idea of what is to come. Similarly, we have reduced some justifications for the simulations performed within the results section, and moved a phrase to the Introduction. Nevertheless, some of them are important to explain the step-by-step logic of what we have done, even if the reasoning was already mentioned in the introduction.

Results:
✓In Figure 2C, the left blue dashed line (indicating the smallest G_+) is not including the G_+ obtained for the Human SC (which is lower). It is not entirely clear if this is a typo or if the authors intend to highlight only the range of G_+ for non-human matrices. In any case, since the orange dashed lines G_- include the Human SC values, it is important to verify this for consistency.

Response: The reviewer is right, there was an inconsistency in the figure. What we intended to highlight was the range for G_- and G_+ in the surrogate connectomes, to highlight the cases where the Human connectome's G_- and G_+ fall outside this range. We have corrected the figure and made the figure legend more explicit.

✓Page 8: 'These heterogeneous activation levels could be distinguished into a low and high activation ranges' -> into low or high activation ranges

Response: Fixed

✓Page 11: ‘Intriguingly, the actual number of ignited nodes at $G = G_-$ is $n = 11$, which is equal to the size of the compact Human s_{max}-core.’ This finding seems quite expected rather than intriguing, so I suggest rephrasing the first word. At least to me, it appears more intriguing at first that the other structures do not show this, but it is of course, plausible.

Response: we have removed the word ‘Intriguingly’.

✓In Figure 5 the ABCD labels are disproportionally large. I suggest reducing their font size.

Response: we have made the size of figures and figure labels more proportional and consistent.

Discussion:

✓In the discussion, I suggest commenting on the fact that some areas never shown ignition in the region of bistability, such as the TP (temporal pole?) which is probably due to the fact that these areas are more distant to the structural core. Also, the way the SC is defined may increase the detection of connections at the core and decrease more distant ones.

Response - The reviewer raises an interesting point. The areas that never ignite correspond to the temporal pole (TP), entorhinal cortex (ENT) and the left parahippocampal cortex (lPARH), which, in our reference *Human* connectome, are also the regions of lowest strength and at the most peripheral shells of the network. These regions continue being peripheral and with the lowest degree even when adopting an unweighed rather than weighed network description of the connectome (with the exception, in this case, of lPARH).
We have added a brief comment about this issue in the Discussion.

Methods:

✓In the equations of the mean field model, there is a typo in the R_i equation (exp exp).

Response: Fixed

Reviewer #2: […]

This is a sound, relevant and very well-written manuscript.

We wish to thank the reviewer for their appreciation of the manuscript.

The authors study the problem of how diverse cortical regions can possibly switch to a state of "ignition" (this term is not my favorite, but seems to be used by some authors) in a transient, dynamical and flexible way.

This term is indeed used in a number of studies by different computational neuroscience authors, all referring to a concept initially brought up in the cognitive neuroscience literature by scholars as Stanislas Dehaene or Raphael Malach and named since then "ignition". Although we agree that this term is referring to technically different phenomena in different studies (see our long section in the Discussion, comparing some of the different notions of "ignition"), we have chosen to use it in order to establish a constructive dialogue with the previous literature.
To avoid any doubt we now explicitly say in the results: "In the context of this study, we use the term "ignition" to refer to switching from the low firing rate baseline state to the higher firing rate state, associated to the existence of dynamic bistability".

[…] This dynamical model, when analyzed in a mean-field setting (e.g. a fully connected network) generates a regime of bistability between what I would call a "down" and an "up" state (baseline of low activity and "ignited" states in the words of the authors). In the language of statistical mechanics, it exhibits a **first-order or discontinuous transition** with a regime of *bistability* and *hysteresis*. […]

We thank the reviewer for pointing out this important terminology that we now explicitly mention in the Introduction, saying: "In the language of statistical mechanics, multistability is due to a discontinuous phase transition associated to hysteresis."

My main question:
✓could the authors show also results for the value of the level of activity (R_{max}, or a better measure of actual network overall activity) at G_- for the HCN as well as for the different randomizations? Is such a value smaller for the HCM than for the randomizations?

Response: We added a new Figure with supplementary data. Figure S15A-B shows the R_{max} value in the range of coupling gain for the Human, uSCs and wSCs ensembles. As a first observation, the value of R_{max} at G_- is always smaller for the human connectome than the surrogate ensembles, except the SW_{hw}. This is not surprising since, given the smaller G_- value, all long-range excitatory inputs received by most regions are weaker. The exceptional behavior of the SW_{hw} could be explained by these ensembles having a lower number of connections in the highest degree nodes, and therefore, a lower integration of the input currents in these nodes. This is not observed in the SW_{rw}. Second, the R_{max} through the whole bistable range is similar between human and uSCs (except in SW_{hw}, for the same reasons discussed above). However, the R_{max} value of the wSCs is higher than the found in the human

in the bistable range. This can be a mixed effect of the number of ignited nodes (always higher in the wSCs than in human) and the weight heterogeneity of the connections of the wSCs ensembles (absent in the uSCs).

I ask this, because the reported phenomenology reminds me very much of that in

Martin, P. V., et al (2015).
Rounding of abrupt phase transitions in brain networks.
J. of Statistical Mechanics: Theory and Experiment, 2015(1), P01003.

Indeed, if I'm not mistaken these authors find that the transition (for a different dynamical model which also exhibits bistability), becomes continuous in the HCN, i.e. the jump of the discontinuous transition vanishes in large networks and becomes much smaller in finite heterogeneous networks. This phenomenology is strongly related to a Griffiths phase as described by some of the same authors above:

Moretti, P., & Muñoz, M. A. (2013).
Griffiths phases and the stretching of criticality in brain networks.
Nature communications, 4(1), 1-10.

After this, the authors scrutinize what are the features of the different cortical regions determining the order in which they are recruited for ignition. The answer is that there is a strong correlation between the rank in the core-shell structure (in a generalization of the standard concept, designed to deal with link strengths rather than with degrees): "All the nodes ignited as early as at the ignition point G_- also belong to the s_{max}-core in the case of the Human. Conversely, all the nodes in the s_{max}-core are ignited already at G_-"

This is, in my opinion the main result of the paper. Maybe it is not extremely surprising but it is nice to see it well documented and well illustrated.

Response: Indeed we find that the phenomena we are describing is well reminiscent of Griffiths phases (even if it is difficult to conclude it rigorously, given how much our small connectome networks are dominated by idiosyncratic finite-size effects and are thus very far from any thermodynamic limit). We have now added a paragraph on the matter to the Discussion, referencing the new Figure S15 mentioned above.

Second request: at this point the reader can be slightly lost as at some point (pag.16) the authors talk about the considered parcellation, but before they have not mentioned that the networks needs to be parcellated nor that this is an important aspect (it might seems trivial, but I think the logical flow would benefit from introducing a discussion of this in the main text).

We have moved a paragraph related to parcellation when the identity of the s_{max}-core regions is described. **"**Remarkably, we obtained similar results with other three empirical connectomes, having different parcellations and network density, that maintain the same relationship (Figs S13 and S14). Although the areas involved are different, they all contain a relatively compact s_{max}-core that is first ignited at a low value of global coupling strength. This

ignition core is also lost when the connectivity-to-weight pattern is disrupted by any randomization procedure.”

The authors show a full set of analyses that I find well-designed and convincing. Also the discussion is very detailed, well structured and thoughtful, pointing out to open questions and future research lines.

Summing up, I think this manuscript is novel, original, well-designed and well-written and it could make a good contribution to PCB.

We wish to thank the reviewer for praising our study.
